# A Comprehensive Biological and Clinical Perspective Can Drive a Patient-Tailored Approach to Multiple Myeloma: Bridging the Gaps between the Plasma Cell and the Neoplastic Niche

**DOI:** 10.1155/2020/6820241

**Published:** 2020-05-18

**Authors:** Antonio Giovanni Solimando, Angelo Vacca, Domenico Ribatti

**Affiliations:** ^1^Department of Biomedical Sciences and Human Oncology, Section of Internal Medicine “Guido Baccelli” University of Bari Aldo Moro, Piazza Giulio Cesare, 11, 70124 Bari, Italy; ^2^Department of Basic Medical Sciences, Neurosciences and Sensory Organs, University of Bari Medical School, Bari, Italy

## Abstract

There is a broad spectrum of diseases labeled as multiple myeloma (MM). This is due not only to the composite prognostic risk factors leading to different clinical outcomes and responses to treatments but also to the composite tumor microenvironment that is involved in a vicious cycle with the MM plasma cells. New therapeutic strategies have improved MM patients' chances of survival. Nevertheless, certain patients' subgroups have a particularly unfavorable prognosis. Biological stratification can be subdivided into patient, disease, or therapy-related factors. Alternatively, the biological signature of aggressive disease and dismal therapeutic response can promote a dynamic, comprehensive strategic approach, better tailoring the clinical management of high-risk profiles and refractoriness to therapy and taking into account the role played by the MM milieu. By means of an extensive literature search, we have reviewed the state-of-the-art pathophysiological insights obtained from translational investigations of the MM-bone marrow microenvironment. A good knowledge of the MM niche pathophysiological dissection is crucial to tailor personalized approaches in a bench-bedside fashion. The discussion in this review pinpoints two main aspects that appear fundamental in order to gain novel and definitive results from the biology of MM. A systematic knowledge of the plasma cell disorder, along with greater efforts to face the unmet needs present in MM evolution, promises to open a new therapeutic window looking out onto the plethora of scientific evidence about the myeloma and the bystander cells.

## 1. Introduction

Multiple myeloma (MM) is an incurable haematological malignancy characterized by a clonal proliferation of plasma cells that accumulate preferentially in the bone marrow (BM). It accounts for 1% of all cancers and 10% of all haematological malignancies. Resistance to chemotherapy poses one of the main challenges in MM management [1]. Indeed, although advances in MM pathophysiological deconvolution and therapeutic knowledge, MM is still an incurable disease [2]. According to Durie–Salmon (D&S) clinical staging, MM patients can be stratified based on available clinical parameters, such as haemoglobin, serum calcium value, X-ray bone study, immunoglobulins, and urine light chains. These parameters may be useful to foresee the patient characteristics from a biological standpoint, in order to predict therapy response and estimate the MM load [3]. Nonetheless, the D&S is affected by observer-related bias in quantifying lytic lesions, and since 2005, it has been replaced by the International Staging System (ISS), which is based only on the combination of two parameters, namely, *β*2-microglobulin and albumin [4]. Nowadays, sensitivity and specificity of bone disease identification have improved, thanks to the widespread use of computed tomography and of functional imaging such as magnetic resonance and positron-emission tomography (PET scan) [1]. Moreover, among the three stages, data on ISS stage III, associated with the poorest outcome, are now available. Since cytogenetics, evaluated by fluorescent in situ hybridization (FISH), is also a major prognostic factor, a new paradigm shift in patient risk stratification has incorporated the three recurrent genetic abnormalities, the t(4; 14), deletion(17p), and t(14; 16), that are mostly associated with a poorer outcome. These are used, along with the clinical and laboratory parameters, in order to gain a more reliable MM risk classification according to the revised ISS (R-ISS) [5]. Undoubtedly, in MM, the genomic landscape has a strong impact on patient outcome and response to therapy [6–8]. Nevertheless, the disease aggressiveness is not only linked to multistep genetic events but also to the MM microenvironment and the MM bystander cells, involved within the tumor niche in a vicious cycle that leads to MM evolution into more complex pathological architecture [9]. BM microenvironment-mediated drug resistance is the main mechanism allowing MM to evade the effects of conventional and new drugs [10]. To date, a plethora of pathophysiological mechanisms has been dissected, but potential targets considered suitable for therapeutic interventions aimed at interfering with the mutual interactions between the clonal plasma cells and the tumor milieu have proven largely unsatisfactory [11]. In this scenario, we have carried out an extensive literature review to probe the novel insights available from translational investigations, in order to reach a deeper understanding of the emerging therapeutic window from a bench-to-bedside standpoint.

## 2. The Role of the Bone Marrow Microenvironment: Novel Molecular Dependencies in Multiple Myeloma

Signals from the bone marrow microenvironment play a pivotal role in supporting MM cell growth, spread, and survival, as well as MM progression [12]. In the bone marrow microenvironment, the cellular compartment consists of hematologic and nonhematopoietic cells such as stromal cells, fibroblasts, osteoblasts, osteoclasts, endothelial cells (ECs), B cells, T cells, natural killer (NK) cells, macrophages, mast cells, and myeloid-derived suppressor cells (MDSCs). During MM development, MM cells can affect the BM cells through cell-cell contact or the secretion of soluble factors to build up a favorable microenvironment. MM cells adhere to bone marrow stromal cells (BMSCs) and trigger many pathways in the latter, resulting in the transcription and secretion of multiple cytokines such as interleukin-6 (IL-6), insulin-like growth factor-1 (ILGF-1), vascular endothelial growth factor (VEGF), and stromal cell-derived factor-1*α* (SDF-1*α*) which mediate MM cell growth, proliferation, survival, and drug resistance [13]. Next, MM cells educate the bone marrow cells to support neoplastic cell growth, survival, and the acquisition of drug resistance resulting in disease relapse, conferring a survival advantage. The tumor microenvironment is recognized as one of the leading factors promoting chemoresistance, but the mechanisms responsible for this effect are still largely obscure [14].

Current studies are focused on the bone marrow microenvironment and inflammatory cells as attractive druggable targets [15]. The MM physiology offers a wide range of targeting opportunities, which can be useful in chemotherapeutics for devising more personalized therapy for MM patients [16]. For example, increased BM hypoxia is associated with increased recirculation of MM plasma cells (MM-PCs). Oxygen delivery decrease, by enhancing hypoxia-inducible factor-2 alpha (HIF-2*α*) activity, induces MM-PC chemokine ligand 12 (CXCL12) upregulation, with a diminished migration toward CXCL12 and reduced adhesion to mesenchymal stromal cells in vitro. HIF-2*α* also strongly induced the expression of chemokine receptor 1 (CCR1) in MM-PCs. CCR1 enhances MM-PC dissemination toward CCL3, while decreasing the MM-PC motility reaction to CXCL12. Additionally, CCR1 upregulation by MM-PCs was correlated with a poor outcome in newly diagnosed MM subjects and associated with enhanced circulating MM-PCs in these individuals. Taken together, these data suggest a role for hypoxia-mediated CCR1 upregulation in driving the egress of MM-PCs from the BM. Targeting CCR1 may be a novel strategy to prevent dissemination and overt relapse in MM [17].

Mesenchymal stem cells (MSCs), one of the main cell components within the BM milieu, can disseminate toward primary tumors and metastatic sites, implying that these cells might modulate tumor growth and metastasis [13]. Myeloma-derived MSCs can deeply impact the disease homeostasis. Therefore, MSCs do not represent bystanders in the BM niche but rather dynamic actors in the MM biology. MSCs can represent a novel target to develop the next generation of therapy in cancer, both by *in vitro* engineering as antitumor carrier to the tumor sites. MM is no exception to this principle [18]. MSCs were lentivirally engineered with osteoprotegerin (OPG) in preclinical models aimed to halt MM-related skeletal lesions [19]. The first-in-class proteasome inhibitor bortezomib shapes the tumor-friendly MM environment by inducing bone matrix remodelling [20] and by interfering with MSC differentiation toward the osteoblastic phenotype [21]. Therefore, combination strategies combined proteasome inhibition with both vitamin D [22] and epigenetic regulators [23]. Building on these strategies, different groups unravelled novel mechanisms able to mobilize and eradicate niche-protected myeloma cells by employing histone deacetylase inhibitors (HDACis) [24]. Pharmacological interfering with nucleosome conformation changes and skeletal metabolism demonstrated the interruption of the molecular crosstalk between MM cells and the stroma and uncovered indirect effects halting cell proliferation, bone disease, and angiogenesis, *in vitro* and *in vivo* [24–26].

The myeloma microenvironment is also characterized by Notch signalling hyperactivation due to the increased expression of Notch 1 and 2 and the ligands Jagged 1 and 2 in tumor cells. Notch activation influences myeloma cell biology and promotes the reprogramming of bone marrow stromal cells. Colombo et al. [27] uncovered Jagged blocking to be relevant for dismal sensitivity to alkylating agents, immunomodulatory drugs (IMiDs), and proteasomal inhibition due to MM cell and tumor milieu-related mechanisms. Enhanced CXCR4/SDF-1 alpha signalling is boosted by Notch overactivation within the MM environment. Additionally, this chemokine axis fuels antiapoptotic mechanisms [27], prompting therapeutic approaches holding the potential to interrupt the vicious cycle between the tumoral PCs and the BMSCs and, conceivably, improve patients' responses to standard-of-care therapies [27].

Furthermore, CXCR4/SDF-1 alpha signalling has been revealed to impact clinical outcome in PC dyscrasias. Nevertheless, treatment strategies pinpointing this receptor or its cognate ligand (burixafor or plerixafor) deemed not adequately proficient. Therefore, a deeper characterization of the biological CXCL12/CXCR4 interaction can offer additional insights, overcoming PC disorder treatment resistance and clonal resilience. This could allow envisioning a novel therapeutic window and a more effective drug combination, designed to halt myeloma progression [28, 29].

Additionally, glycosylation, by modulating different aspects of tumor biology, can be considered as a hallmark of cancer. Several solid and haematological malignancies are characterized by enhanced sialylated glycan expression, with a direct correlation with higher disseminated behaviour. Sialylation can also prime MM homing into its environment by physical interaction between skeletal precursors, stromal cells, and MM cells creating niches and educating bone cells. Therefore, interfering with sialylation may promote translational navigation of the milieu-drug resistance boundaries and define alternative combinatorial treatment strategies bringing sialylation inhibitors to the MM-stroma interface [30]. Thus, nanotechnologically engineered tools provided next-generation strategies for tailored anti-MM therapy by optimization of pharmacokinetics and pharmacodynamics profile of conventional chemotherapeutic agents [31]. In detail, novel anthracycline preparation integrating integrin *α*4*β*1 within nanoparticles seems to be able to exert enhanced anti-MM and dismal off-target effects, offering a proof of concept of the value of this pharmacokinetics innovation [31]. An alternative approach was delivering liposomal formulation carrying combinations of taxanes, alendronate, and isoform-adapted transferrin, enabling microenvironment drug modulation [32]. Notably, novel engineered tagging strategies combined modern immunotherapeutic targeting with either proteasome inhibitor [33] or bone-modifying agents (BMAs) [34], gaining more efficient off-target profile. Specifically, CD38 receptor- and B-cell maturation antigen- (BCMA-) directed approaches have introduced a practice change in immunotherapy and are being intensively investigated in MM [34, 35], since these molecules are highly expressed on the malignant plasma cells.

Sialylation inhibition using these approaches also promises incremental activity in interfering within the MM-niche vicious cycle. Recently, sialyltransferase inhibitors restored affective anti-MM activity by restoring innate and acquired immune response, while halting malignant cell proliferation at the same time [36].

Currently, delivery systems employing the sialylation inhibitor 3Fax-Neu5Ac encapsulation in combination with BMA are intensively investigated, in order to potentially block MM homing and enhance drug efficacy as well. In frame of this thinking, Natoni et al. [30] have shown that BMSCs can nurse MM by shaping an immune-tolerogenic milieu and uncovered sialylation as an actionable mechanism to boost the immune response [30].

### 2.1. Angiogenesis in Multiple Myeloma

In 1994, Vacca et al. [37] demonstrated for the first time that bone marrow microvascular density (MVD) was significantly increased in MM compared to monoclonal gammopathies of undetermined significance (MGUS) and even more in active vs. nonactive forms. The close association between angiogenesis and active MM indicates that it is the vascular phase of plasma cell tumors. Conversely, MGUS and nonactive MM represent the avascular phase. The microvessel area and the labeling index (LI) percent are closely associated with the MM activity phase and are mutually correlated [38–41].

In 2011, Ria and colleagues [15] also highlighted neovascularization as a constant hallmark of MM progression. This process is only partially supported by factors such as VEGF, fibroblast growth factor-2 (FGF-2), and metalloproteinases (MMPs), which are directly secreted by the tumor cells. As a consequence of plasma cell-stromal cell interactions, the cytokines within the MM niche, in particular IL-6, drive the release of angiogenic factors from bystanders in the bone milieu, these being one of the main triggers of the angiogenic switch during disease progression. But along with angiogenesis, vasculogenesis also occurs in the tumor niche of MM subjects, enhancing the vascular tree formation. In the neoplastic microenvironment of MM individuals, hematopoietic stem cells are primed to become ECs by the angiogenic cytokines shed in autocrine, paracrine, and endocrine fashion.

Therapeutic strategies in MM consist of conventional chemotherapy and biologically based therapy targeting not only MM-PCs but also the microenvironment and angiogenesis. Bortezomib regulates many cellular processes, including the modulation of transcription factors, such as NF-*κ*B, cell cycle progression, inflammation, immune surveillance, growth arrest, and apoptosis. NF-*κ*B is a major transcriptional factor which mediates the expression of many proteins including cytokines, chemokines, and cell adhesion molecules. Bortezomib inhibits NF-*κ*B, enhancing the susceptibility of MM plasma cells to therapeutics, while the induction of IL-6 by BMSCs mediated by NF-*κ*B increases the secretion of VEGF from MM-PCs. Furthermore, bortezomib inhibits MMEC mitotic activity, through inhibition of VEGF, IL-6, insulin-like growth factor-1 (IGF-1), and angiopoietin-1 and angiopoietin-2 (Ang-1 and Ang-2) [42].

The circulating levels of Ang-1, Ang-2, VEGF, and angiogenin were measured in 54 patients with smouldering MM (SMM). This result was compared with those of 27 MGUS patients, 55 MM patients, and 22 healthy controls, demonstrating that the ratio of circulating Ang-1/Ang-2 was reduced in MM individuals with full-blown overt MM due to a biologically significant enhancement of Ang-2, but not in SMM or MGUS nonmalignant control subjects. VEGF and angiogenin were increased in all patients compared to controls. However, circulating VEGF was higher in symptomatic MM compared to SMM and MGUS, while angiogenin was reduced. Hence, the above data show that the Ang-1/Ang-2/Tie-2 axis may be an effective target for the development of novel antimyeloma agents [43]. Bortezomib downregulates not only the caveolin-1 tyrosine phosphorylation, responsible for VEGF-mediated MM cell migration, but also the caveolin-1 phosphorylation induced by VEGF in ECs. Finally, bortezomib inhibits the transcription of ICAM-1, VCAM1, and E-selectin [44].

Thalidomide is an antiangiogenic drug. It modulates tumor necrosis factor-alpha (TNF-*α*) signalling through direct and indirect effects on the tumor microenvironment [45]. It also reduces FGF-2 [46], VEGF, and IL-6 secretion by BMSCs and MM cells [47], stimulating the activation and expansion of T cells and enhancing NK-cell-mediated cytotoxicity. Thalidomide disrupts the host marrow-MM cell interactions by selectively modulating the density of cell surface adhesion molecules. Nonetheless, treatment with thalidomide induces side effects while lenalidomide and pomalidomide, its derivatives, are both less toxic and more potent [48, 49]. Cereblon (CRBN) is a primary target of thalidomide teratogenicity, but it is also required for the antimyeloma activity of thalidomide and related drugs (IMiDs). A decreased CRBN expression is linked to pharmacological resistance in human MM cell line models and primary cells and may also provide a biomarker to predict IMiD response and resistance [49]. In fact, other authors analysed the influence of the single-nucleotide polymorphisms (SNPs) of the CRBN gene on the risk of adverse effects of thalidomide-based chemotherapy in patients with MM [50].

However, Curry and colleagues found no reduction in MVD before or after treatment with thalidomide of newly diagnosed MM (NDMM) patients [51]. Nevertheless, other authors showed that high MVD at diagnosis was considered an independent poor prognosis factor [52].

Lamanuzzi and colleagues [53] evaluated mTOR activation in ECs from 20 patients with MGUS and 47 patients with MM and its involvement in angiogenesis. mTOR and the rapamycin-insensitive companion of mammalian target of rapamycin (RICTOR), two components of mTORC2 complex [54], were deemed significantly elevated in MMECs compared to MGUS-ECs. The authors uncovered mTORC2 expressed by MMECs to be relevant for angiogenic boosting and found that mTOR/RICTOR targeting by siRNA and dual mTOR inhibitor PP242 reduced the MMEC angiogenic functions, including cell migration, chemotaxis, adhesion, invasion, *in vitro* angiogenesis on Matrigel®, and cytoskeleton reorganization. Additionally, in the chick embryo chorioallantoic membrane (CAM) and in Matrigel® plug assays, PP242-directed approaches demonstrated angiogenic blockade *in vivo* by interfering with angiogenesis. PP242 exerted a synergistic effect with IMiDs and proteasome inhibitor, suggesting that mTOR blockade can enhance the antiangiogenic effect of these drugs. Because mTORC2 involved in MM angiogenesis, dual mTOR inhibitor PP242 could support antiangiogenic management of MM patients [53].

Bisphosphonates exert a direct effect on MM plasma cells [55]. In detail, both zoledronic acid and neridronate have a cytotoxic activity on tumor cells and inhibit angiogenesis [55–57]. The side effect is osteonecrosis of the jaw (ONJ), a long-lasting disorder that occurs mainly in breast cancer and MM patients treated with intravenous bisphosphonates [58].

Recently, the role of CX3CL1/fractalkine has been reported, as a novel mechanism of this cell signalling boosting angiogenesis and inflammation in multiple myeloma. ADAM10 and ADAM17 are responsible for cleavage and shedding, thereby modulating CX3CL1/fractalkine release. Notably, these MMPs are regulated by inflammatory and angiogenesis processes. Firstly, assessment of soluble levels in plasma cell disorders at different disease stages demonstrated that circulating concentration of CX3CL1 was significantly higher in full-blown disease compared with controls [59]. Strikingly, this observation was correlated with BM microvessel density. Next, ensuing functional *in vitro* experiments recapitulated fractalkine dynamics, highlighting the theragnostic role of enhanced production of this chemokine by MM-derived BM endothelium upon exogenous stimulus [59, 60]. In fact, Tanaka et al. [61] uncovered mAb-blocking strategies anti-CX3CL1 as next-generation approach aimed at halting innate and adaptive immune-dependent inflammation. Finally, Chen et al. also corroborated this evidence demonstrated decreased CX3CL1 production in vivo, upon proteasome inhibition [62]. These compelling data envision also the use of anti-TNF-*α*, in combination with the abovementioned therapeutic strategies for MM patients [59].

### 2.2. Endothelial Cells

MMECs express VEGF/VEGFR-2, FGF-2/FGF-2 receptor-2 (FGF-2R-2), and Ang-2/Tie-2 and exert an increased *in vitro* and *in vivo* angiogenic activity [63]. Moreover, MMECs express CXC chemokines CXCL8/IL-8, CXCL11/interferon-inducible T-cell alpha chemoattractant (I-TAC), CXCL12/SDF-1*α*, and CCL2/monocyte chemotactic protein-1 (MCP-1), which mediate plasma cell proliferation and homing [64].

Long-term MMECs were compared with MGUSECs and HUVECs as their normal quiescent counterpart [63]. MMECs but not healthy cells overexpressed endothelial activation markers [65]. Mechanistically, MMECs increasingly produce FGF-2, VEGF, MMP-2, and MMP-9 compared to HUVECs, conferring a growth advantage over controls by a faster establishment of a proangiogenic phenotypic behavior, in terms of capillary sprouting and net formation [65]. MMECs also boost a strong proangiogenic response in the CAM [66]. Gene expression assays corroborated these pieces of evidence, uncovering MMECs' phenotype to be characterized by enhanced proangiogenic gene transcription, namely, VEGF, FGF isoforms, HGF, Tie2, transforming growth factor-beta (TGF-*β*), GRO-*α* chemokine, fibronectin-1, HIF-1*α*, ETS-1, ID3, and osteopontin compared to HUVECs.

MMECs alone displayed a VEGF-dependent autocrine growth loop [65], owing to high VEGF and VEGFR-2 expression, constitutive autophosphorylation in both VEGFR-2 and the associated kinase ERK-2, along with the inhibition of proliferation, capillarogenesis, and phosphorylation by neutralizing anti-VEGF and anti-VEGFR-2 antibodies. Pentraxin 3 affected MMEC functional activities and was able to modify the angiogenic capability of both MMECs and plasma cells [67].

Comparative gene expression profiling was made of MMECs and MGUSECs with Affymetrix U133A arrays [68]. Expression of 22 genes deemed significantly different by comparing MMECs with MGUSECs. Key biological processes related to protumorigenic functions were affected, showing significant gene expression deregulation in the symptomatic disease when compared to the precursor's states. Next, *DIRAS3*, *SERPINF1*, *SRPX*, *BNIP3*, *IER3*, and *SEPW1*, gene-encoding proteins, were functionally tested to substantiate the gene signature findings, corroborating their proangiogenic function in BMECs. *BNIP3*, *IER3*, and *SEPW1* transient gene silencing had a significant impact on programmed death, cell proliferation, adhesion, and angiogenesis-related functions. Four proteins were found to be overexpressed in MMECs: filamin A, vimentin, *α*-crystallin B, and 14-3-3*ζ*/*δ* protein [69]. Their expression was enhanced by VEGF, FGF-2, HGF, and MM-PC-conditioned medium and their silencing RNA knockdown affected MMEC angiogenesis-related functions, including spreading, migration, and tubular morphogenesis [69].

More recently, Leone described a novel aspect of disease pathophysiology, by characterizing the MM cell interface with the local environment, namely, vascular endothelium between ECs and CD8^+^ lymphocyte, create a permissive immune microenvironment within the BM, allowing plasma cell proliferation. In this context, the corrupted endothelium behaves as tolerogenic promoter, by indirect negative regulation of the effector memory CD8^+^ T cells. The CD8^+^ T-cell population sustained by ECs also expressed Foxp3, producing IL-10 and TGF-*β*, and exerting a protumorigenic activity. The above study adds further insight into the role that ECs play in MM biology and describes an additional immune regulatory mechanism that inhibits the development of antitumor immunity and may impair the success of cancer immunotherapy [70].

Extracellular vesicles (EVs) shed from the MM cell surface actively participate in cellular crosstalk and vessel formation during MM progression [71]. Proteasome inhibition via Bi-EVs decreased EC proteasome activity, and Bi-EVs released from apoptotic MMECs promoted angiogenesis suppression by decreasing the proliferation and migration of ECs [71] IMiDs exerted a relevant antiangiogenic effect *in vivo,* and *in vitro*, it also inhibited migration of MMECs, but not of MGECs or control HUVECs. VEGF/VEGFR-2 cell signalling was deemed biologically connected to lenalidomide treatment, which exerted a significant impact on cytoskeleton rearrangement, migration, and cell metabolism in MMECs [72].

### 2.3. Macrophages and Mast Cells

Tumor-associated macrophages and mast cells support tumor growth and neovascularization by producing a wide array of angiogenic cytokines. Mast cell- and macrophage-derived growth factors that can promote tumor development and angiogenesis include TNF-*α*, TGF-*β*1, FGF-2, VEGF, platelet-derived growth factor (PDGF), IL-8, osteopontin, and nerve growth factor (NGF). Conversely, mast cell- and macrophage-produced cytokines that may participate in antitumor responses include IL-1, IL-2, IL-4, IL-10, and interferon-gamma (IFN-*γ*) [73].

When BM macrophages from MM patients are exposed to VEGF and FGF-2, they transform into cells that are functionally and phenotypically similar to paired MMECs, and generate capillary-like networks mimicking those of MMECs [74]. Macrophages from nonactive MM, MGUS, and benign anaemia patients display similar albeit weaker features. EC-like macrophages and apparently typical macrophages contribute considerably to the formation of new vessels in patients with active MM, whereas their vascular supply is minimal in nonactive MM and absent in MGUS patients and control patients [74]. In contrast to MGUS and asymptomatic disease, CD14/CD68 surface overexpression has been found in full-blown myeloma. BM trephine immune staining additionally dissected two macrophage subpopulation, demonstrating cells with either endothelial or conventional phenotype by CD68/FVIII-RA coloration. Remarkably, proangiogenic effects on macrophages have shown to contribute to the building of neovessel wall in patients with active MM over nonactive and MGUS conditions [74].

Proinflammatory macrophages in BM biopsies are a potential prognostic biomarker for acquired MM resistance to bortezomib therapy, and high levels in BM are correlated with poor survival. Remarkably, proteasome inhibitor treatment of proinflammatory macrophages primed MM-tumor-initiating cell (MM-TIC) infiltration both *in vitro* and *in vivo* in an IL-1*β*-dependent fashion. One way to abolish bortezomib-induced MM-TIC enrichment is by blocking the IL1*β* axis using a pharmacologic or genetic approach [75]. Additionally, CD163 expression was detected by immunohistochemistry to determine the number of tumor-associated macrophages (TAMs) in 198 MM patients receiving bortezomib-based regimens. Enhanced CD163+ TAM infiltration in NDMM was correlated with a worse clinical outcome, in terms of progression-free survival (PFS), overall survival (OS), and a worse therapy response quality compared with subjects with lower CD163+ TAM infiltration. These data indicate that the CD163+ TAM content at diagnosis is a powerful predictor of prognosis in MM [76]. Another link between the effect of bortezomib in MM patients and macrophages was highlighted by Khalife's studies. They demonstrated the improved treatment effectiveness gained by miR-16 increased expression in boosting anti-MM activity by a proteasome inhibitor in the presence of MM resident TAMs [77]. Enhanced soluble miR-16 in MM individuals linked to more favourable outcome. Conversely, deletion 13 on cancer cells was inversely associated with peripheral miR-16 concentration [78]. miR-16 can be actively secreted by MM cells through EVs, with a direct correlation between intracellular and shed levels. EVs isolated from MM patients can drive circulating monocyte differentiation to M2-TAMs, while the increased concentration of circulating miR-16 reverts this behaviour. *In vivo*, miR-16 lost sustains macrophage differentiation toward an M2 phenotype acquisition, most likely due to dismal NF-*κ*B activation *via* IKK*α*/*β* targeting [77]. Moreover, the immune function of macrophages is mediated by IL-32*γ*, which is overexpressed in MM patients and associated with a more advanced clinical stage [79]. Gene expression profiling showed a significant IL-32*γ*-dependent induction of the immunosuppressive molecule indoleamine 2,3-dioxygenase (IDO) in macrophages, and this effect was verified by qRT-PCR, western blotting, and immunofluorescence. Proteinase 3 (PR3), an IL-32 binding protein, was universally expressed on the surfaces of macrophages, and PR3 knockdown or the inhibition of the STAT3 and NF-*κ*B pathways hindered the IL-32-gamma-mediated stimulation of IDO expression. These results indicate that MM cell-derived IL-32*γ* promotes the immunosuppressive function of macrophages and is a potential target for MM treatment [80].

MGUS and smouldering disease seem to be characterized by a peak of mast cell density count-related and angiogenesis enhancement [81]. Ang-1 is a crucial promoter of MM cell growth by stimulating angiogenesis. Experimental evidence indicates that Ang-1 secreted by primary murine mast cells promotes marked neovascularization in an *in vivo* transplantation assay [82]. Primary mast cells accelerate tumor growth by established plasmacytoma cell lines, while Ang-1-neutralizing antibodies significantly reduced the growth of plasmacytomas containing mast cells. Moreover, mast cell infiltrate parallels proangiogenic cytokine concentration, growth-related oncogene-alpha (GRO-alpha), and epithelial neutrophil-activating protein-78 (ENA-78). The authors also demonstrated that mast cell density was correlated with ki-67 PI, suggesting an important participation of mast cells in MM biology and growth [83]; in this context, mast cells would enhance angiogenesis, produce cytokines with growth effects on myeloma cells, and modify the BM microenvironment [84]. Therefore, mast cells could be indicators of the disease activity [85] and valuable targets for therapeutic interventions [83]. In line with this viewpoint, mast cells may be a novel target for an adjuvant strategy aimed at halting angiogenesis by interrupting the vicious cycle underlying cytotoxic cytokine production, thus circumventing mast cell-mediated immune suppression [86].

BM specimens from active myeloma over premalignant derived trephines analysed by both laser and electron microscopy were characterized by the presence of neovessels lined by granulated mast cells [87]. Otherwise, in MGUS, mast cells are localized on the abluminal side of neovessels [87]. However, mast cell density has an impact not only on angiogenesis but also on the progression of bone disease in MM patients. In 52 MM patients, BM mast cell density was measured by immunohistochemical staining for tryptase, and serum levels of MMP-9 and RANKL were measured by a solid-phase sandwich enzyme-linked immunosorbent assay. Additionally, urinary N-terminal propeptide of procollagen type I (Ntx) concentrations was assessed by an enzyme-linked assay, at different disease stages and bone involvement. Enhanced mast cell count, RANKL, and Ntx concentrations were found in MM subjects. Furthermore, mast cell density was positively correlated with MMP-9, RANKL, and Ntx. Therefore, mast cells may contribute to osteolytic processes during MM progression [88].

### 2.4. Cancer-Associated Fibroblasts (CAFs) in MM

In the progression of the disease from MGUS to MM, a fibroblast switch is required to acquire protumorigenic activity and parallels the behaviour exhibited by other haematological and solid cancers [89–92]. The switch was demonstrated by the bone marrow fibroblast gene expression profile of patients with MGUS and MM, extracted by nonnegative matrix factorization (NMF) [93]. Moreover, a specific miR profile in BM fibroblasts is linked to the transition from the asymptomatic to the full-blown disease. BM fibroblasts and EV-dependent vicious cycle orchestrated by MM cells determine an enhanced production of miR-27b and miR-214, fuelling proliferative and antiapoptotic pathways. These prosurvival functions parallel an increased expression of fibroblast activation markers alpha-smooth muscle actin (*α*SMA) and fibroblast activation protein (FAP). While strengthening the mechanisms involved in the transition from MGUS and SMM to MM, a peculiar miRNA profile in MM-associated fibroblasts, along with the myeloma cells, educates the BM microenvironment by priming the BM fibroblast phenotype [94]. In fact, Desantis et al. studied the effect of recombinant human erythropoietin (rHuEPO) on MM fibroblasts *in vivo* and *in vitro*. It had previously been demonstrated that rHuEPO regulated angiogenic responses in MM via a direct effect on macrophages and ECs. Likewise, rHuEPO decreases the activation marker (*α*SMA and FAP) expression in MGUS and MM; furthermore, proinflammatory and proangiogenic cytokines, such as IL-6 and IL-8, VEGF-A, FGF-2, and HGF in MM fibroblasts, significantly diminished. Collectively, rHuEPO halted the MM-associated fibroblast proliferation. Conversely, fibroblast-programmed cell death enhanced in both MGUS and MM. Overall, these data pinpoint rHuEPO as a key brake on MM-supporting fibroblast action [95].

### 2.5. Myeloid-Derived Suppressor Cells

MDSCs are myeloid cells with a specific inhibitory activity on the immune response, which accumulate in the tumor microenvironment during tumor development [96] Significant accumulations of immunosuppressive MDSCs were observed in the BM of patients at early stages of MM and regulated MM growth by inhibiting T cells [97]. Moreover, murine MM cells directly activate BM MDSCs and enhance their immunosuppressive function through soluble factors such as granulocyte-macrophage colony-stimulating factor (GM-CSF), promoting the immune escape of MM cells [98]. An increase of bone marrow MDSCs was also detected in the 5T33 MM mouse model after inoculation with MM cells [98]. In the BM of 5T33 MM mice, exosomes derived from MM cells increased the number of BM MDSCs *in vivo* and induced changes in MDSC subpopulations which are similar to their phenotype, suggesting the involvement of exosomes in the accumulation of MDSCs [99].

MDSCs can mediate the suppression of myeloma-specific T-cell responses through the induction of T-cell anergy and Treg development in the MM microenvironment [100]. Polymorphonuclear (PMN)-MDSCs and neutrophils equivalently sustain MM resistance to alkylators and doxorubicin, by mediating soluble factor production. Targeting PMN-MDSCs could enhance chemotherapy efficacy in MM. It is well accepted that targeting MDSCs in cancer improves the immune response and increases the efficacy of immunotherapy. MDSCs play an ancillary role as a suitable target to overcome MM drug resistance, an important finding in light of recent data suggesting the benefit of combined chemo- and immunotherapy treatment protocols [100–102]. Due to the loss of equilibrium in the MM immune landscape, immune checkpoint targeting agents have not shown clinical activity in MM. It is therefore critically important to deal with immunosuppressive mechanisms and improve immune responses, especially in advanced MM patients. New immunotherapeutic strategies such as immunomodulatory drug-intensified monoclonal antibody treatment, immune checkpoint inhibitors, and chimeric antigen receptor T-cell therapy targeting B-cell maturation antigen have been employed in advanced-stage MM patients [103]. An association between high PMN-MDSC levels and poor overall survival in MM patients has been validated. PMN-MDSCs induced piRNA-823 upregulation, which in turn enhanced DNA methylation, thus stimulating the MM cell clonal evolution. Silencing of piRNA-823 in MM cells reduced the stemness of MSCs maintained by granulocytic (G)-MDSCs, resulting in a decreased tumor burden and angiogenesis *in vivo* [104]. It has also been demonstrated that the proinflammatory cytokine IL-18 is critically involved in MM and its levels are associated with MDSCs. IL-18-deficient mice were remarkably protected from MM progression in a CD8^+^ T-cell-dependent manner. Within the BM milieu, IL-18 stimulates MDSCs, sustaining MM progression. High levels of BM plasma IL-18 were associated with poor survival in MM patients. The above preclinical studies suggested that IL-18 could be a potential therapeutic target in MM [105]. Additionally, the estrogen effect in hematologic malignancies including MM was studied, and treatment with 17beta-estradiol significantly promoted the progression of the disease. However, this effect has not been attributed to a direct effect of estrogen on MM cells but was considered to be mediated through estrogen-induced alterations in the tumor microenvironment. In particular, it significantly increased the ability of MDSCs to suppress T-cell proliferation [106].

Botta et al. [107] pointed out developments in MDSC-directed approaches, by suggesting applications toward histone-deacetylase inhibitors in MM and uncovered signalling pathways involving MDSCs [108, 109], able to halt inflammation, by impairing JAK/STAT downstream. Epigenetic modulation reduced cell ability of monocytic phenotype granulocytic shift, by promoting macrophages or dendritic cells shaping within the tumor niche and opening novel therapeutic windows [110–112]. All-trans retinoic acid (ATRA) indeed enhanced MAPK activation with dismal reactive oxygen species levels, prompting mature myeloid lineage fuelling [113, 114].

Despite the existence of a correlation between MDSC pathophysiology and proangiogenic factors, VEGF-blocking mAb strategies did not succeed. Furthermore, the likelihood of MDSC-induced reduced sensitivity to the antiangiogenetic therapy discouraged further attempts in this direction [115]. Conversely, promising results generated by investigating miRNA-based approaches [112, 116] hold the potential to reduce MM disseminated potential and provided the bases to reveal MDSC-related targets to identify, mobilize, and eradicate niche-protected cells likely able to favor MM progression [117].

Aiming to implement MDSC-dependent immunosuppression halting strategies, several attempts have been made to interfere with cyclooxygenase-2 (COX-2), arginase-1 expression, and inducible nitric oxide synthases and to decrease reactive oxygen species production and provided undeniable rational for the novel association of anti-inflammatory compound to the MM therapeutic backbone, in order to expand the effectiveness of immunotherapy and to decrease the myeloid-derived population in the MM environment [118]. Remarkably, the antigen-presenting cell (APC) capacity of dendritic cells and ECs [76] can also open a further therapeutic window, since several examples have recently been published, highlighting the tight crosstalk between the immune microenvironment gene signature, vascular cells, and molecular targets, in both haematological and solid cancers [119, 120]. These studies point to the WNT pathway as a druggable, theragnostic marker with a plethora of effects on the immune microenvironment in cancer [121].

This complex scenario fostered an intensive translational investigation aimed at improving MM immune equilibrium lost via MDSC targeting. Other noteworthy aspects are related to the immune microenvironmental landscape and its modulation through fluoropyrimidine, nucleoside analogues, and anthracyclines [122]. Nonetheless, state-of-the-art development [123, 124] holds great potential in circumventing myeloid-derived immune suppression by interfering with critical signals, such as IL-4 receptor *α* (IL-4R*α*), thus reducing cell proliferation. Moreover, the binding of the aptamer to its specific receptor led to MDSC depletion and tumor growth. Peptide enrichment in both M- and G-MDSCs by phage-dependent strategies led to the development of a peptibody, via the fusion of peculiar peptide sequences with the Fc portion of murine IgG2b monoclonal antibodies, demonstrating *in vivo* activity [124].

**Figure 1:**
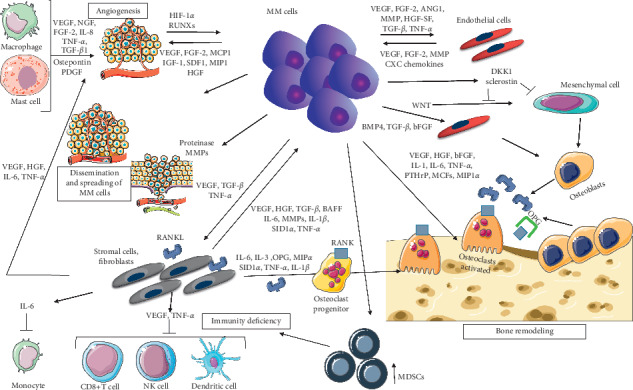
Soluble factors and adhesion-related interactions between MM cells and bone marrow immune microenvironment: MM-PCs prime the tumoral milieu via a plethora of mechanisms. Immune cell function, by deficiency in adaptive and innate response dysfunction, and proinflammatory cytokine production drive essential signals for microenvironment colonization and interactions. Moreover, MM-PCs control neoplastic survival and dormancy, modulating the response of the BM microenvironment cells to MM dissemination. Bone disease in MM is a prototypical malignant bone microenvironment pathologic condition. By tackling the knowledge gap on skeletal dissemination and disruption and cell-cell and cell-matrix interaction, the prevention and cure of MM progression may be better understood and targeted by immunomodulation, using combinations of MM-PC-directed agents against novel therapeutic targets.

The main complexities of the MM microenvironment cell network are summarized in Figure 1.

Multiple myeloma, MM; vascular endothelial growth factor, VEGF; nerve growth factor, NGF; fibroblast growth factor, FGF; interleukin, IL; tumor necrosis factor-alpha, TNF-*α*; transforming growth factor-beta 1, TGF-*β*1; platelet-derived growth factor, PDGF; hypoxia-inducible factor-1 alpha, HIF-1*α*; runt-related transcription factor, RUNXs; monocyte chemotactic protein 1, MCP 1; insulin-like growth factor 1, IGF-1; stromal cell-derived factor 1, SDF-1; MIP1; angiopoietin 1, ANG1; metalloproteinases, MMP; hepatocyte growth factor/scatter factor (HGF/SF), chemokine, CXC; Dickkopf, DKK; wingless-type MMTV integration site family, WNT; bone morphogenetic protein-4, BMP4; parathyroid hormone-related protein, PTHrP; macrophage colony-stimulating factors, MCFs; B-cell activating factor, BAFF; SID1 transmembrane family, member 1, SID1; receptor activator of nuclear factor-kappa-Β ligand, RANKL; osteoprotegerin, OPG; natural killer, NK; myeloid-derived suppressor cells, MDSCs.

## 3. Therapeutic Windows

A new goal in haematological malignancies is represented by a treatment approach targeting not only patients with active MM, but also those with SMM. This shift toward early intervention [125] with the antiangiogenic agents lenalidomide and dexamethasone demonstrated prolonged disease-free survival and OS in patients with SMM.

## 4. Targeting Angiogenesis and the Immune Microenvironment in Multiple Myeloma: Current Challenges

The knowledge of critical pathways supporting angiogenesis and creating immunosuppression during MM evolution uncovered a reciprocal crosstalk between MM cells with the surrounding milieu, and compelling verification designates that angiogenesis and immunosuppression often fuel a simultaneous vicious cycle [70]. Consequently, approaches relating to antiangiogenic immune mechanisms seem to hold the promise to tip the equilibrium of the MM environment and increase clinical benefit. The first-in-class drug thalidomide and its derivative lenalidomide mirrored the abovementioned knowledge, representing one possible translation from bench-to-bedside efforts [51, 126]. But as stated above, the precise target of lenalidomide is cereblon, since low cereblon levels are associated with drug resistance [127–129]. Undeniable evidence supports the use of drugs that target the BM microenvironment to prevent the progression of SMM or full-blown MM. Additionally, in a mouse model, the use of an antiangiogenic anti-VEGFR-2 antibody in the early stage delayed tumor progression of MM; nonetheless, besides IMiDs, angiogenic-directed strategy did not show effective results in the unselected patient subgroup in patients with MM [130]. In order to achieve a patient-tailored vasculogenic targeting, stringent patient stratification has been proposed by modulating from the critical step of MM evolution that can be critically dependent on vessel supply, such as smouldering phases [131] or extramedullary dissemination [132–134].

The next breakthrough of therapeutic strategy design is targeting the MM ecosystem together with the immune microenvironment. The altered BM niche sustains the proliferation of MM cells, nursed by physical and soluble reciprocal interactions educating both the neoplastic and the immune environmental cells [135]. Identification, mobilization, and eradication of this niche-protected dormant and often pharmacological insensitive cells have been significantly improved since several trials involving antibodies have proved clinical benefits in MM. Anti-SLAMF7 targeting by elotuzumab anti-CD38-directed approaches by daratumumab as a single agent or with proteasome inhibitor and IMiDs have shown far more effectiveness and superior activity than the standard of care [136, 137]. The first-in-class SLAMF7 targeting molecule stimulates NK cells and macrophages; conversely, CD38-targeting by daratumumab induces the immune system triggering toward Treg reduction and by enhancing T-helper and cytotoxic lymphocytes [138]. Another target studied especially in melanoma, lung cancer, and Hodgkin lymphoma is programmed cell death 1 (PD-1) [139, 140]. In more detail, the pieces of evidence that PD-1/PD-ligand 1 (PD-L1) modulation increases T- and NK cell antimyeloma effects prompted the use of immune checkpoint inhibitors in clinical studies. Nevertheless, the anti-PD-1/PD-L1 mAb as a single agent did not provide sufficient results. Lack of infiltrating effector cells within the MM milieu can explain the modest efficacy demonstrated by these clinical trials [141]. Therefore, drug combination strategies encouraged clinical trials (NCT02289222 and NCT02331368), uncovering encouraging medical response [142, 143]. In addition, studies of chimeric antigen receptor-T-cell (CAR-T-cell) therapy targeting BCMA have revealed very high response rates in heavily pretreated patients with MM [140, 144].

As pointed out, CXCL12 and its ligand CXCRA can have a pathologic role in different stages of MM and patient drug resistance, so disrupting the CXCL12-CXCR4 axis might be a therapeutic opportunity [39]. Roccaro et al. uncovered CXCL12 and CXCR4 as putative targets to halt MM evolution and extramedullary dissemination in animal models [28, 145], indicating broad potential consequences on adhesion-mediated MM dissemination [29, 101, 146] and drug resistance, as in other solid and haematological malignancies [147–149] and prompt clinical validation [150]. Therapeutic interventions with burixafor or plerixafor (CXCR4 antagonists) in MM are not efficient enough [39].

Bisphosphonates (pamidronate or zoledronic acid) [56] and other BMAs, such as DKK1 inhibitors (Dickkopf WNT signalling pathway inhibitors), antisclerostin mAb [151], and RANKL inhibitors (denosumab) [152], represent an example of attempts to target the disseminated and localized bone disease effect due to the spreading of malignant plasma cells. Nonetheless, despite encouraging clinical outcome gained in full-blown disease [153], to date no clinical evidence demonstrated a survival benefit by treating the asymptomatic version of myeloma [154].

**Figure 2:**
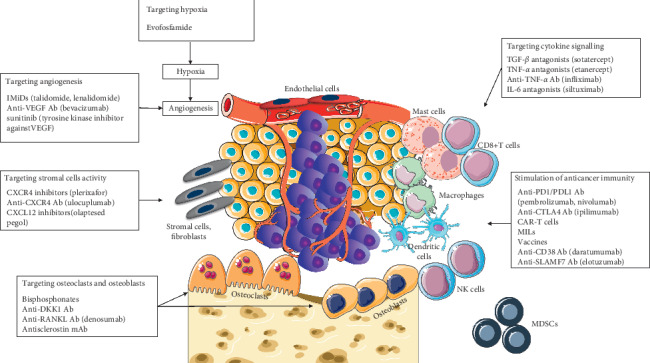
Therapeutic targets within the tumor milieu in multiple myeloma. Immunomodulatory drugs, IMiDs; vascular endothelial growth factor, VEGF; antibody, Ab; chemokine receptor 4, CCR4; chemokine ligand 12, CXCL12; Dickkopf, DKK; receptor activator of nuclear factor-kappa-Β ligand, RANKL; transforming growth factor-beta 1, TGF-*β*1; tumor necrosis factor-alpha, TNF-*α*; interleukin-6, IL-6; programmed cell death 1/programmed cell death ligand 1, PD-1/PD-L1; chimeric antigen receptor-T cells, CAR-T cells; marrow infiltrating lymphocytes, MILs; self-ligand receptor of the signalling lymphocytic activation molecule, SLAMF7.

Studies conducted on MM cell lines have shown that the addition of exogenous IL-6 is essential for obtaining the growth of neoplastic cells *in vitro*. By removing IL-6 from short-term cultures, MM cells die, demonstrating that this cytokine acts as both a growth factor and a survival factor. In long-term and high cell density cultures, malignant plasma cells become progressively independent and are able to produce IL-6 as an autocrine growth factor [155]. These pieces of evidence prompted several investigations aimed at characterizing additional soluble substances supporting MM in the environment. TGF-*β* is one of the best candidates deserving druggable intervention investigation [156], employing several blocking approaches [157, 158], demonstrating clinical activity in the treatment of MM-associated bone diseases. Luspatercept treatment has been shown to have a potential impact on MM-related kidney involvement (Table 1 and Figure 2).

Ultimately, adequate oxygen pressure is essential for proper physiologic conditions and insufficient hypoxia is a conspicuous characteristic in various physiological and pathological processes, including neoplastic disorders and cancer dissemination [159]. In MM, increased BM hypoxia is associated with an increased recirculation of neoplastic cells [160], leading to loss of pharmacological sensitivity and priming resistance to radiotherapy [161]. Therefore, alkylators selectively activated by insufficient oxygen supply condition were tested in preclinical models [162] and in clinical trials involving patients with MM [162, 163], showing an effective inhibition of HIF-1-alpha in MM both alone and in combination [164]. Intriguing results from phase I/II clinical studies results supported further investigation in relapsed/refractory subjects [163]. The therapeutic opportunity window and pathophysiological aspects are summarized in Figure 2.

## 5. Conclusions

MM is likely one of the hematologic conditions in which the major advances from biology to new therapy have occurred over the last years. The biology outlook has shifted from morphology and basic biochemical analysis to an integrated multi-Omics approach offering novel therapeutic perspectives. Nonetheless, MM finally progresses to a relapse/refractory stage, levying a heavy impact on patient survival and quality of life. The MM microenvironment pathophysiologic determinants, defined from a validated prognostic perspective, provide clinicians with novel insight, offering the potential to deal with the unmet medical need for prolonged, sustained disease remission. The interactions of multiple myeloma cells with different subsets of immune cells and ECs within the BM tumor niche environment seem to be the ideal backbone supporting the ultimate translation of biological findings into improved diagnostics and therapies.

## Figures and Tables

**Table 1:** Compounds in advanced investigation targeting MM cells and the tumor microenvironment.

Targets	Therapeutic agents
Angiogenesis	(1) IMiDs (thalidomide^*∗*^, lenalidomide^*∗*^)
	(2) Anti-VEGF Ab (bevacizumab)
	(3) Tyrosine kinase inhibitor against VEGF (sunitinib)

Hypoxia	(1) Evofosfamide investigational hypoxia-activated prodrug

Stromal cells	(1) CXCR4 inhibitors (plerixafor)
	(2) Anti-CXCR4 Ab (ulocuplumab)
	(3) CXCL12 inhibitor (olaptesed pegol)

Osteoclasts and osteoblast	(1) Bisphosphonates^*∗*^
	(2) Anti-DKK1 Ab
	(3) Anti-RANKL Ab (denosumab^*∗*^)
	(4) Antisclerostin mAb

Cytokine signalling	(1) TGF-*β* antagonists (sotatercept)
	(2) TNF-*α* antagonists (etanercept)
	(3) Anti-TNF-*α* Ab (infliximab)
	(4) IL-6 antagonist (siltuximab)

Stimulation of anticancer immunity	(1) Anti-PD-1/PD-L1 Ab (pembrolizumab, nivolumab)
	(2) Anti-CTLA4 Ab (ipilimumab)
	(3) CAR-T cells
	(4) MILs
	(5) Vaccines
	(6) Anti-CD38 Ab (daratumumab^*∗*^, isatuximab)
	(7) Anti-SLAMF7 Ab (elotuzumab^*∗*^)

Immunomodulatory drugs, IMiDs; vascular endothelial growth factor, VEGF; antibody, Ab; chemokine receptor 4, CCR4; chemokine ligand 12, CXCL12; Dickkopf, DKK; receptor activator of nuclear factor-kappa-Β ligand, RANKL; transforming growth factor-beta 1, TGF-*β*1; tumor necrosis factor-alpha, TNF-*α*; interleukin-6, IL-6; programmed cell death 1/programmed cell death ligand 1, PD-1/PD-L1; chimeric antigen receptor-T cells, CAR-T cells; marrow infiltrating lymphocytes, MILs; self-ligand receptor of the signalling lymphocytic activation molecule, SLAMF7. ^*∗*^FDA and EMA approved.
